# Descriptive epidemiology of external structural birth defects in Enugu State, Nigeria

**DOI:** 10.4314/gmj.v56i4.5

**Published:** 2022-12

**Authors:** Izuchukwu F Obi, Ugochukwu U Nwokoro, Okechukwu P Ossai, Michael I Nwafor, Patrick Nguku

**Affiliations:** 1 Nigeria Field Epidemiology and Laboratory Training Programme, Asokoro, Abuja, Nigeria; 2 Department of Community Medicine, University of Nigeria Teaching Hospital Ituku-Ozalla, Enugu State, Nigeria; 3 Hospitals Management Board, Enugu State, Nigeria; 4 Department of Obstetrics and Gynaecology, University of Nigeria Teaching Hospital Ituku-Ozalla, Enugu State, Nigeria

**Keywords:** Occurrence of birth defects, Congenital anomalies, Congenital malformations, Overt structural birth defects, Enugu State Nigeria

## Abstract

**Objectives:**

To determine the birth prevalence, trend, and characteristics of external structural birth defects occurrence in Enugu Metropolis, Nigeria.

**Design:**

Cross-sectional study involving review of delivery records.

**Setting:**

The study was conducted at three tertiary hospitals, one public and two missionary, in Enugu Metropolis.

**Participants:**

Mothers and their babies delivered between 1 January 2009 and 31 December 2016 in the study facilities.

**Main outcome measures:**

Birth prevalence of defects presented as frequency/10,000 births. Other descriptive variables are presented as frequencies and percentages.

**Results:**

There were 21530 births with 133 birth defects (birth prevalence: 61.8/10,000 births) and 1176 stillbirths (stillbirth rate: 54.6/1000 births). The frequencies and birth prevalence (/10,000 births) of recorded defects were: Limb deformities 60(27.9), Neural tube defects (NTDs): 36(16.7), Urogenital system defects: 12(5.6), Gastrointestinal system defects 10(4.6) and Orofacial clefts 4(1.9). Birth defects occurrence showed a rising trend from 2009 to 2016. The mean (SD) age of mothers whose babies had Birth defects was 29.1(4.7) years. Only 62(46.6%) of 133 antenatal clinic folders of these women were traceable for further review. Eighteen (29.0%) had febrile illness in early pregnancy, 9(14.5%) had Malaria, 17(27.4%) had <4 antenatal clinic attendance, 7(11.3%) did not take folic acid and 6(9.7%) took herbal medications during pregnancy.

**Conclusions:**

Birth defects occurrence showed a rising trend with limb deformities and NTDs having the highest prevalence. Record keeping was poor at the facilities. Birth defects preventive interventions like folic acid supplementation for women-of-childbearing age should be promoted in Enugu Metropolis.

**Funding:**

This work was supported by the non-communicable disease Minigrant from the Task Force for Global Health, Decatur, Georgia, USA (TPN-FE-NCD-C2-IFO-9).

## Introduction

Birth defects are important public health problems which contribute significantly to infant and childhood disability and deaths.^1^ Globally, at least 3.3 million children under five years of age die annually from complications of serious birth defects, with 3.2 million survivors being at risk of permanent mental or physical disability.^2^ Birth defects account for 25.3–38.8 million disability-adjusted life-years (DALYs) worldwide 3 and 303,000 neonates are also estimated to die within four weeks of birth due to birth defects.^1^

Although birth defect is a global problem, its impact is particularly severe in low- and middle-income countries (LMICs). Over 94 per cent of births with serious defects and 95 per cent of deaths in affected children occur in LIMCs due to high fertility rates and high prevalence of birth defects.^2^

Robust surveillance systems are necessary for effective planning, evaluation and control of birth defects. Unfortunately, national surveillance of birth defects remains limited worldwide.^4^

In an effort to promote global birth defects surveillance, the World Health Assembly in 2010 issued a resolution urging member states to develop and strengthen registration and surveillance systems for birth defects.^5^ Despite this global call, evidence show that most countries, especially those in sub-Saharan Africa, do not have surveillance systems for birth defects. A systematic review of reports from birth defects surveillance registries and peer-reviewed publications, between January 1990 and July 2014, on the occurrence of neural tube defects (NTDs) worldwide, showed that only 75 (38.7%) of 194 WHO member states had eligible publications or reports. Africa and South-East Asia had the least available data with only 8 (17%) of 47 African member states reporting.^4^ The general absence of birth defect registries and surveillance systems coupled with the paucity of research on birth defects in most sub-Saharan African countries make it difficult to estimate the true prevalence of birth defects in this region.

Studies from different sub-Saharan African countries have reported birth prevalence of congenital defects varying from 0.4 to 28.1 cases per 1000 births.^6^,^7^ Furthermore, some hospital based studies conducted in different parts of Nigeria reported birth prevalence of congenital defects varying between 3.5 and 63 cases per 1000 births.^8^–^13^ Some of these studies extracted data from delivery records 8,12,13, paediatrics records 10,11 or both.^9^ Majority of studies on the prevalence of birth defects in Nigeria focused on selected system defects like central nervous system defects (9.8 cases per 1000) 14, orofacial defects (0.5 to 13.5 cases per 1000) 15,16 and congenital cardiac defects (0.5 cases per 1000).^17^ So far, all the studies on birth defects in Enugu State, Nigeria have been in government-owned hospitals with majority focusing only on one system defects.^14^,^18^–^20^ Yet, evidence reveal that out of the 86.9% of deliveries in the state which occur in healthcare facilities, majority (56.9%) take place in private facilities and only 27.3% in government-owned facilities.^21^ Nonetheless, no study was found to have reported on the occurrence of birth defects in private facilities in the state. We determined the birth prevalence and characterised the occurrence of external structural birth defects among babies delivered in selected private and government-owned healthcare facilities in Enugu metropolis from 2009 to 2016.

## Methods

### Study area and setting

The study was conducted in three tertiary healthcare facilities (two organised private/ missionary hospitals and the state government-owned teaching hospital) located in Enugu Metropolis, the capital city of Enugu State in Southeast geopolitical zone of Nigeria. Enugu state has 17 local government areas (LGAs); five urban and twelve rural. Three urban LGAs (Enugu North, Enugu South and Enugu East LGAs) make up Enugu Metropolis. The year 2020 projected population of Enugu Metropolis is 779,879 with women of childbearing age constituting 20% of this population.^22^ Igbos are the predominant ethnic group in Enugu State constituting over 92% of residents with Hausa, Fulani, Yoruba and other minority ethnic groups making up the remaining 8%.^22^ The total fertility rate of women in the state is 3.8 births per woman.^23^

The government-owned teaching hospital and one of the missionary hospitals are in Enugu North LGA while the other missionary hospital is in Enugu south LGA. These three facilities, among other services, offer specialist medical, surgical, emergency, delivery, paediatric and immunisation services. They receive referrals from other lower cadre healthcare facilities in the state. The labour wards in these facilities maintain surveillance data on birth defects occurrence unlike most of the lower cadre hospitals. Two of the selected facilities have high delivery rates (each has an average of 150 deliveries per month) while one of the missionary hospitals have an average of 60 deliveries per month. There is neither surveillance system nor registry for birth defects in Enugu State.

### Study design and population

We conducted a cross-sectional study involving review of labour ward delivery registers for all deliveries conducted in three study facilities over an eight-year period (January 1, 2009 to December 31, 2016). For infants identified to have external structural birth defect(s), the mothers' clinic folders were traced and reviewed for more information.

### Sample size and sampling procedure

A whole population study was conducted in three specialist healthcare facilities in Enugu Metropolis purposively selected because they were the only facilities in the metropolis that capture data on the occurrence of birth defects in their delivery registers. The records of all deliveries conducted in these selected facilities from 2009 – 2016 were reviewed.

### Data collection

The selected facilities keep hard copy delivery registers which contain the following information: patient name, age, folder number, address, booking status, date and time of delivery, mode of delivery (vaginal or abdominal), parity, live or stillbirth, baby's sex, birth weight, Apgar score and description of birth defects if any.

These facilities also have antenatal/ postnatal clinic folders in which detailed patient information were captured. The extent of information captured in these folders varies within and between healthcare facilities (depending on the rigor of attending healthcare worker).

Case ascertainment was from diagnosis and verbatim case description documented by unit specialist doctors and nurses with or without archived pictures.

### Instrument and data collection procedures

The researcher and four trained research assistants used a pre-tested aggregate data abstraction sheet (S1) and proforma (S2) adapted from the WHO/CDC/ICDBSR Birth Defects Surveillance Manual and other similar studies^7^,^13^,^24^,^25^ to abstract relevant data from delivery registers and maternal folders respectively. The aggregate data abstraction sheet had the following sections: Health facility identification, year of report, month, number of births, live birth and stillbirths for each month, foetal sex, total number with birth defects and number with different birth defects. The proforma used to abstract data from the folder of mothers of babies with birth defect(s) captured the following information: foetal and parental characteristics, description of identified birth defect(s) and maternal risk factors.

### Data analysis

Data were entered and analysed with Microsoft Excel spreadsheet and Epi Info version 7.1. The occurrence of birth defects was summarised as frequencies, percentages and birth prevalence (incidence) computed thus:

Birth prevalence of birth defects = (total number of birth defects cases/ Total births) x 10,000

The trend of birth defects occurrence over eight years was illustrated with a line graph and the characteristics of babies and the mothers of babies with birth defects were described and summarised with frequencies and percentages.

### Ethical considerations

Ethical approval was obtained from the Human Research Ethics Committee of Enugu State Ministry of Health (reference: CON/MHPHD/1772/84). Informed consent was not sought for this study because it involved retrospective review of anonymised surveillance data, however consent to review treatment records was obtained from the hospital authorities and all retrieved data were de-identified.

### Operational definitions

External structural birth defect(s) are anatomical congenital defects identifiable by health workers by observation or through physical examination of neonate as routinely carried out immediately after delivery without necessarily conducting diagnostic investigations.

## Results

During the 8 years review period, 21,530 births were recorded in the three study facilities with 133 birth defects (prevalence: 61.8/10,000 births) and 1176 stillbirths, giving a stillbirth rate of 54.6/1000 births. Ninety-eight (73.7%) of the babies with defects were born in the state teaching hospital out of the 11,155 (51.8%) deliveries that took place there. (Table 1)

**Table 1:** Distribution of birth outcomes by Health Facility, Enugu, Nigeria, 2009–2016

Variables	Healthcare Facility			Total
		
	State Teaching Hospital	Missionary Hospital 1	Missionary Hospital 2	
**Total births**	11155	9313	1062	21530
**Live births**	10434	8883	1037	20354
**Number with** **birth defects**	98	32	3	133
**Birth prevalence** **of Birth** **defects** **(/10,000** **births)**	87.9	34.4	28.2	61.8
**Number of** **Still Births**	721	430	25	1176
**Stillbirth** **rate (/1000** **births)**	64.6	46.2	23.5	54.6

The mean ±SD birth weight of babies with defects was 3.1 ±1.1 kg, the median (IQR) gestational age at birth was 38.0 (36.0, 40.0) completed weeks and majority 78 (58.7%) of these babies were males. The mean ±SD age of mothers whose babies had birth defects was 29.1 ±4.7 years and majority 129 (97.7%) of them were of the Igbo ethnic group. (Table 2)

**Table 2:** Characteristics of babies with birth defects in Enugu, Nigeria, 2009 – 2016

Variables N=133	Frequency (%)
**Foetal sex**	
**Male**	78 (58.7)
**Female**	49 (36.8)
**Ambiguous**	4 (3.0)
**Unclassified**	2 (1.5)
**Birth weight in kg (N=124)** *	
**Mean ±SD**	3.1 ±1.1
**< 2.5**	39 (31.4)
**≥ 2.5**	85 (68.6)
**Gestational age (completed weeks)**	
**Median (IQR)**	38.0 (36.0, 40.0)
**Birth Outcome**	
**Livebirth**	100 (75.2)
**Fresh stillbirth**	26 (19.5)
**Macerated stillbirth**	7 (5.3)
**Maternal age in years (N=119)** *	
**Mean ±SD**	29.1 ±4.7
**≤ 30 years**	77 (64.7)
**> 30 years**	42 (35.3)
**Parental consanguinity (N=125)** *	
**No**	123 (98.4)
**Unknown**	2 (1.6)
**Maternal Ethnicity (N=132)** *	
**Igbo**	129 (97.7)
**Hausa**	2 (1.5)
**Yoruba**	1 (0.8)

* Variable incomplete for all 133 cases

Limb deformities had the highest birth prevalence of 27.9/10,000 births, followed by neural tube defects (NTDs) with a prevalence of 16.7/10,000 births. Among the NTDs, Anencephaly had the highest prevalence of 6.5/10,000 births while Polydactyly had the highest prevalence (18.1/10,000 births) among the limb deformities.

The prevalence of Urogenital system defects was 5.6/10,000 births while that of Gastrointestinal defects and Orofacial clefts were 4.6 and 1.9/ 10,000 births respectively. Six babies (2.8/10,000 births) had Down syndrome while four had unclassified birth defects. (Table 3)

**Table 3:** Distribution of recorded birth defects in Enugu, Nigeria, 2009–2016

Type of Birth defect	Frequency (%)	Birth Prevalence (/10,1000 births)
**Limb deformities**	60* (45.1)	**27.9**
**Polydactyly**	39 (29.3)	18.1
**Talipes equinovarius**	8 (6.0)	3.7
**Achondroplasia**	4 (3.0)	1.9
**Talipes genuvalgus**	2 (1.5)	1.0
**Amelia**	2 (1.5)	1.0
**Neural tube defects**	36* (27.1)	**16.7**
**Anencephaly**	14 (10.5)	6.5
**Hydrocephalus**	11 (8.3)	5.1
**Spina bifida**	7 (5.3)	3.3
**Encephalocele**	1 (0.8)	0.5
**Orofacial clefts**	**4 (3.0)**	**1.9**
**Gastrointestinal System** **defects**	**10 (7.5)**	**4.6**
**Imperforate anus**	4 (3.0)	1.9
**Omphalocele**	4 (3.0)	1.9
**Gastrochisis**	2 (1.5)	1.0
**Urogenital system**	**12 (9.0)**	**5.6**
**Ambiguous genitalia**	4 (3.0)	1.9
**Hypospadias**	4 (3.0)	1.9
**Undescended testes**	3 (2.3)	1.4
**Epispadias**	1 (0.8)	0.5
**Down syndrome**	**6 (4.5)**	**2.8**
**Conjoined twins**	**1 (0.8)**	**0.5**
**Unclassified defects**	**4 (3.0)**	**1.9**
**Total Birth defects**	**133 (100.0)**	**61.8**

* Birth Defects under this subgroup did not add up to the group total because some defects were captured in the register with the subgroup name.

**Figure 1:**
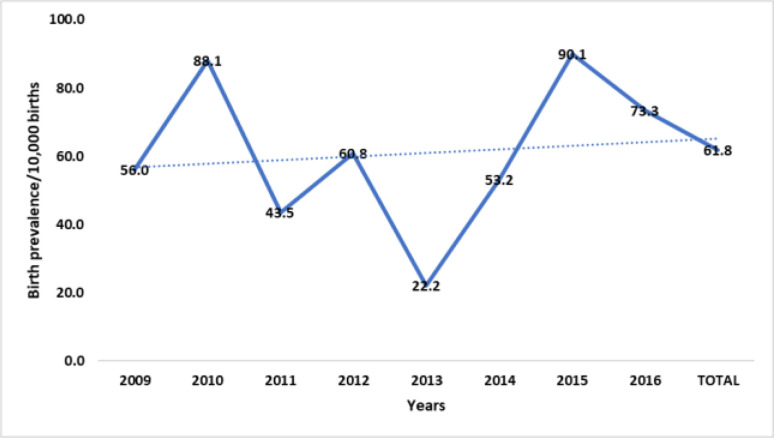
Trend of birth defects occurrence over 8 years, Enugu, Nigeria, 2009-2016

The overall trend of annual birth defects prevalence in the state was that of a slow but progressive rise, with undulating birth prevalence between 2009 and 2016. Two peaks (88.1 and 90.1/10,000) occurred in 2010 and 2015 respectively, with a significant dip (22.2/10,000) in 2013. (Figure 1)

Only 62 out of the 133 folders of mothers of babies with birth defects were traceable for further analysis. Eighteen (29.0%) of these mothers had fever in the first trimester, half of which were confirmed to be due to Malaria, 17 (27.4%) did not attend antenatal clinic up to four times before delivery and 7(11.3%) did not take folic acid during the index pregnancy. Six (9.7%) women took herbal medications while 9 (15.5%) took alcohol during the index pregnancy. (Table 4)

**Table 4:** Characteristics of mothers of babies with birth defects, Enugu, Nigeria (N=62)

Characteristics of mothers	Number of mothers exposed* (%)
**History of febrile illness in first trimester**	18 (29.0)
**Less than 4 antenatal clinic attendance in** **pregnancy**	17 (27.4)
**History of confirmed Malaria in first trimester**	9 (14.5)
**Alcohol intake during pregnancy**	9 (14.5)
**Chronic maternal disease before/ during** **pregnancy**	8 (12.9)
**No folic acid intake during pregnancy**	7 (11.3)
**Took herbal medications during pregnancy**	6 (9.7)
**Exposed to ionising radiations during pregnancy**	1 (1.6)
**Cigarette smoking during pregnancy**	1 (1.6)
**History of birth defects in previous pregnancies**	1 (1.6)
**Took teratogenic drugs during pregnancy**	1 (1.6)
**Vaccination against Toxo, Rubella etc.**	0 (0.0)

* Some mothers had multiple exposures

## Discussion

This study determined the birth prevalence and characterised the occurrence of external structural birth defects among babies delivered selected private and government-owned healthcare facilities in Enugu metropolis from 2009 to 2016. The overall birth prevalence of external structural birth defects was 61.8/10000 births.

The prevalence in the two private (missionary) hospitals (28.2 and 34.4 cases /10000 births) were much lower than the prevalence in the government-owned teaching hospital (87.9/10000). The higher prevalence observed in the teaching hospital could be because it receives most referrals of high-risk pregnancies from the lower-level primary healthcare facilities and maternity homes in the state. Moreover, the cost of maternal and child health services in the teaching hospital is subsidised by the government, thereby attracting more pregnant women in the low-income group whose living conditions might increase their likelihood of exposure to teratogenic substances. Indeed, low-income have been shown to be an indirect determinant of congenital defects.^1^ The overall prevalence of 61.8 birth defects per 10000 births found in this study is similar to the incidence reported by another study in Enugu metropolis which involved review of delivery records.^12^ It is also similar to the prevalence reported by a population-based study in southern Vietnam 26 and another facility-based study in Iran.^27^ On the contrary, the March of Dimes estimated prevalence of 735/1000 livebirths for Nigeria 2 and the prevalence of 282/10000 livebirths from a facility-based study in Northern Nigeria 28 are both higher than our finding. The use of live births as denominator in these two studies as against total births in ours may have contributed to the higher prevalence reported by these studies. Nevertheless, a facility-based study in south-south Nigeria 13 and another involving review of admissions in a neonatal ward of a tertiary facility in Northern Nigeria 10 reported lower prevalence of 35.3 and 36.5/10000 births respectively.

In our study, limb deformities had the highest prevalence followed by neural tube defects (NTDs). Polydactyly, a minor limb deformity, was the single most frequent birth defect while anencephaly was the most common neural tube defect. The high prevalence of NTDs found in our study is disturbing because this group of defects are largely preventable through pre-natal screening, pre-conception and pre-natal folate supplementation in women of childbearing age.^29^ Pre-conception folate supplementation and pre-natal screening for congenital abnormalities are rarely practiced in Nigeria. Although the Maternal and Child Health Unit of Enugu State Ministry of Health promote the use of periconceptional folate supplementation through public awareness and health education campaigns, studies in the state^14^ and other states in Nigeria^30^ have shown low levels of periconceptional folate supplementation. In most cases, there is usually non-use of pre-conception folate supplementation and late commencement of folate supplementation by pregnant mothers during booking visits, which usually occur in the second trimester.^31^

Similar to the finding in our study, some studies assessing the prevalence of birth defects in different parts of Nigeria and other sub-Saharan African countries have reported anomalies of the musculoskeletal system 8,13,32 or limb deformities 7,26 to be the most prevalent birth defects. On the other hand, some studies conducted in northern Nigeria 9,10,28 and other parts of Africa 33,34 reported NTDs as the most common group of congenital anomalies. Some of these studies focused on major external structural defects thereby excluding polydactyly, a minor defect, from the limb deformities group. Likewise, excluding polydactyly from the limb deformities group in our study will make NTDs, the most common group of deformities.

Anencephaly was the most common NTD in our study, followed by hydrocephalus and spina bifida. Sadly, almost half of the babies in the NTDs group had Anencephaly, a condition with an estimated mortality of 100%, thus increasing the perinatal and neonatal mortality rates in this area. Likewise, a cross-sectional study involving review of one-year delivery records in a teaching hospital in Accra Ghana, reported Anencephaly to have the highest prevalence (8.4/10000 births) followed by spina bifida.^35^ Also another facility-based study in Iran reported Anencephaly as the most common NTD.^36^ On the contrary, a hospital-based study 14 conducted in a tertiary facility in Enugu State Nigeria and another 18 in a tertiary facility in Port Harcourt Nigeria, reported spina bifida as the predominant NTD. The study population in these two studies were babies admitted in the New-born Special Care Units (NBSCU) unlike our study that reviewed the records of deliveries in the labour ward. Because anencephaly usually results in stillbirth, most anencephalic babies do not make it to the NBSCU of hospitals, explaining why spina-bifida was the most common NTD in these studies.

Moreover, orofacial clefts had a prevalence of 1.9 cases per 10000 births in our study. Babies with orofacial clefts usually have problems with feeding, speaking or hearing.^37^ The prevalence in our study is lower than the finding in two studies 13,38 involving a review of delivery registers in other parts of Nigeria. The dissimilar findings in these studies may be due to differences in geographical location. Furthermore, a multi-centre study involving review of data from seven orofacial clefts treatment centres in six geopolitical zones of Nigeria,^15^ also reported higher prevalence of orofacial clefts than our study. Referral bias may have contributed to the higher prevalence found in the multi-centre study.

This study further revealed a rising trend of birth defects in the metropolis, with a sharp increase noticed from 2013 (22.2/10000 births) to 2015 (90.1/10000 births) with only a small decrease (73.3/10000) in 2016. The rising birth prevalence of defects could be a pointer to increasing levels of potentially harmful exposures among women of childbearing age in our environment. About 15% of women whose babies had birth defects in our study reported taking alcohol during the index pregnancy, while 10% reported taking herbal concoctions. Raising awareness about birth defects and the need to minimise exposure to avoidable risk factors among adolescent girls and women of childbearing age is important in our environment.

There was male preponderance (M: F ratio of 1.6:1) among babies with birth defects in our study. Several studies 8,10,12,14 in different parts of Nigeria have also reported male preponderance in birth defects occurrence. On the other hand, while some studies 28,39 reported foetal low birth weight to be a risk factor for birth defects, the majority (68.6%) of babies with birth defects in our study had normal birth weights. Unfortunately, our study design did not permit testing for association between foetal birth weight and occurrence of birth defects. The mean age of mothers whose babies had birth defects was 29.1 years, with about 65% of these mothers being 30 years of age or younger. This finding agrees with other studies 33,36 which found majority of babies with birth defects being born to women within the age range 21 to 30 years. However, a study in Benghazi reported higher occurrence of birth defects among babies born to mothers above 40 years.^40^ Although parental consanguinity has been shown to be a risk factor for birth defects 36,39, none of the babies with birth defects in our study had consanguineous parents as such marriages are culturally unacceptable in our environment.

### Limitations

Our study, being hospital-based, may have under-ascertained the occurrence of birth defects in our environment compared to population-based studies which give better estimates. Furthermore, data completeness was a challenge in this study as about half of the antenatal clinic folders of mothers whose babies had birth defects could not be found for analysis. Moreover, information captured in maternal folders and delivery registers varied across facilities as there was no uniform surveillance tool available in the state. The above limitations notwithstanding, this study happens to be the first to assess the occurrence of birth defects in private healthcare facilities where most pregnant women in the state are known to give birth.

## Conclusion

The overall birth prevalence of external structural birth defects in Enugu Metropolis was 61.8 cases per 10000 births; with higher rates in the government-owned tertiary hospital compared to the missionary hospitals. Musculoskeletal and Neural tube defects (NTD) had the highest birth prevalence with anencephaly being the most common NTD. Majority of babies with birth defects had normal birth weight while one-in-four of such babies were stillborn. Record keeping was poor at the facilities and some of the mothers whose babies had birth defects were exposed to potential risk factors during pregnancy. We recommend establishment of birth defects surveillance system and population-based registries to enable more robust estimation of the prevalence of birth defects in the state. Birth defects preventive measures including folic acid supplementation among women of childbearing age should also be promoted in the state.
